# Impact of the Spanish Smoke-Free Legislation on Adult, Non-Smoker Exposure to Secondhand Smoke: Cross-Sectional Surveys before (2004) and after (2012) Legislation

**DOI:** 10.1371/journal.pone.0089430

**Published:** 2014-02-27

**Authors:** Xisca Sureda, Jose M. Martínez-Sánchez, Marcela Fu, Raúl Pérez-Ortuño, Cristina Martínez, Esther Carabasa, María J. López, Esteve Saltó, José A. Pascual, Esteve Fernández

**Affiliations:** 1 Tobacco Control Unit, Cancer Control and Prevention Programme, Institut Català d'Oncologia-ICO, L'Hospitalet de Llobregat, Barcelona, Spain; 2 Cancer Control and Prevention Group, Institut d'Investigació Biomèdica de Bellvitge-IDIBELL, L'Hospitalet de Llobregat, Barcelona, Spain; 3 Department of Clinical Sciences, School of Medicine, Universitat de Barcelona, L'Hospitalet del Llobregat, Barcelona, Spain; 4 Biostatistic Unit, Department of Basic Science, School of Medicine and Health Sciences, Universitat Internacional de Catalunya, Satn Cugat del Valles, Spain; 5 Bioanalysis Research Group, Neuropsychopharmacology Programme, IMIM-Hospital del Mar Research Institute, Parc de Recerca Biomèdica de Barcelona, Barcelona, Spain; 6 Catalan Network of Smoke-free Hospitals, Insitut Català d'Oncologia, L'Hospitalet de Llobregat, Barcelona, Spain; 7 Department of Nursing, School of Medicine and Health Sciences, Universitat Internacional de Catalunya, Sant Cugat del Valles, Spain; 8 Evaluation and Interventions Methods Service, Agència de Salut Pública de Barcelona, Barcelona, Spain; 9 Biomedical Research Centre Network for Epidemiology and Public Health (CIBERESP), Agència de Salut Pública de Barcelona, Barcelona, Spain; 10 Institut d'Investigació Biomèdica-IBB Sant Pau, Barcelona, Spain; 11 Public Health Agency, Ministry of Health, Generalitat de Catalunya, Barcelona, Spain; 12 Department of Public Health, School of Medicine, Universitat de Barcelona, Barcelona, Spain; 13 Department of Experimental and Health Sciences, Universitat Pompeu Fabra, Parc de Recerca Biomèdica de Barcelona, Barcelona, Spain; Universität Bochum, Germany

## Abstract

**Background:**

In 2006, Spain implemented a national smoke-free legislation that prohibited smoking in enclosed public places and workplaces (except in hospitality venues). In 2011, it was extended to all hospitality venues and selected outdoor areas (hospital campuses, educational centers, and playgrounds). The objective of the study is to evaluate changes in exposure to secondhand smoke among the adult non-smoking population before the first law (2004-05) and after the second law (2011–12).

**Methods:**

Repeated cross-sectional survey (2004–2005 and 2011–2012) of a representative sample of the adult (≥16 years) non-smoking population in Barcelona, Spain. We assess self-reported exposure to secondhand smoke (at home, the workplace, during leisure time, and in public/private transportation vehicles) and salivary cotinine concentration.

**Results:**

Overall, the self-reported exposure to secondhand smoke fell from 75.7% (95%CI: 72.6 to 78.8) in 2004-05 to 56.7% (95%CI: 53.4 to 60.0) in 2011–12. Self-reported exposure decreased from 32.5% to 27.6% (−15.1%, p<0.05) in the home, from 42.9% to 37.5% (−12.6%, p = 0.11) at work/education venues, from 61.3% to 38.9% (−36.5%, p<0.001) during leisure time, and from 12.3% to 3.7% (−69.9%, p<0.001) in public transportation vehicles. Overall, the geometric mean of the salivary cotinine concentration in adult non-smokers fell by 87.2%, from 0.93 ng/mL at baseline to 0.12 ng/mL after legislation (p<0.001).

**Conclusions:**

Secondhand smoke exposure among non-smokers, assessed both by self-reported exposure and salivary cotinine concentration, decreased after the implementation of a stepwise, comprehensive smoke-free legislation. There was a high reduction in secondhand smoke exposure during leisure time and no displacement of secondhand smoke exposure at home.

## Introduction

Exposure to secondhand smoke (SHS) has been causally associated with many adverse health effects[1]. Worldwide, it has been estimated that, in 2004, exposure to SHS was responsible for 379,000 deaths due to ischemic heart disease, 21,400 deaths due to lung cancer, 165,000 due to lower respiratory infections, and 36,900 due to asthma[2]. In Spain, between 1228 and 3237 deaths due to lung cancer and ischemic heart diseases have been attributed to SHS exposure[3].

Exposure to SHS can occur in different settings, including in the home, at the workplace, in other private and public places (bars, restaurants, cafes, etc.), and inside public and private transport vehicles. Questionnaires, biomarkers, and airborne markers have been used to evaluate SHS among non-smokers. The prevalence of SHS exposure in adult non-smokers varies considerably, depending on the country, the development of the tobacco epidemic[4], the comprehensiveness of smoke-free legislation, and the location of exposure to SHS. Worldwide, 33% of male non-smokers and 35% of female non-smokers were exposed to SHS in 2004[2]. In Spain, 75% of the adult non-smoking population was exposed to SHS in 2006; of those, 26.4% was exposed at home and 39.8% at work or an educational venue[5]. In Barcelona, in the period of 2004–2005, the prevalence of self-reported exposure to SHS among non-smokers in all settings was similar to that of the whole country[6].

On the 1^st^ of January, 2006, a smoke-free legislation (Law 28/2005) was implemented in Spain to protect the health of non-smokers. The legislation banned smoking in all public and work places, with some exceptions in hospitality venues (no ban in venues of less than 100 m^2^, and ‘smoking areas’ were allowed in venues over 100 m^2^)[7]. Some previous studies evaluated the impact of that law and showed important reductions in the exposure to SHS at the workplace[8], but no significant changes occurred either at home or during leisure time[9]; furthermore, and importantly, exposure to SHS was not reduced in bars or restaurants[8], [10], [11]. Due to the evidence provided by those evaluations, and after intensive advocate work, the law was amended[12]. On the 2^nd^ of January, 2011, a new legislation (Law 42/2010) was established to amend Law 28/2005. The new Spanish legislation extended the smoking ban to all hospitality venues (bars, cafes, pubs, restaurants, discos, and casinos) without exception,[13] and extended the ban to some outdoors areas, including hospital premises, educational campuses, and playgrounds. The law included economic penalties for infringements and its enforcement is a responsibility of the regional and local health authorities. After the implementation of the new law, SHS levels (measured as the quantities of airborne nicotine and PM2.5) have decreased more than 90% in hospitality venues[14], [15]. However, the impact of the more restrictive smoke-free legislation has not been assessed for SHS exposure in the general population.

The objective of this study was to evaluate whether a measurable change in SHS exposure could be detected in the adult non-smoking population with the implementation of the stepped Spanish smoke-free legislation. We compared SHS exposure measurements (self-report data and levels of salivary cotinine) before the first law (2004–05) and after the second law (2011–12) legislation.

## Methods

### Study design and selection of study participants

This study had a repeated cross-sectional design. We included a representative, random sample of the population of Barcelona (Spain). Surveys were conducted before and after the implementation of smoke-free legislation. The pre-legislation data were obtained between March 2004 and December 2005. We used the same strategy to collect the post-legislation data between June 2011 and March 2012. Detailed information about the pre-legislation survey (sampling, face-to-face questionnaire, saliva collection, and cotinine analysis) has been provided in previous studies[6], [16].

In brief, for each survey, we determined a sample size of 1,560 people with standard procedures (α error of 5%, beta error of 20%, and 20% losses for independent samples). The pre-legislation survey (years 2004–05), included a final sample of 1,245 individuals and the post-legislation survey included a final sample of 1,307 individuals. These sample sizes were sufficient to detect 10% changes in the amount of exposure to SHS at the workplaces or at home (under the least favorable conditions) and a 40% difference in salivary cotinine concentrations between the two surveys. Sample size calculations were performed with 5.2 GRANMO MS Windows (http://www.imim.es/media/upload/arxius/grmw52.zip).

We obtained data and addresses for Barcelona residents from the updated official city census (years 2001 and 2010) provided by the Municipal Institute of Statistics of Barcelona. Individuals aged 16 years and older were eligible to participate in the study. A letter was mailed to eligible individuals to inform them about the purpose of the study and that they had been selected at random. The letter also informed them that the study required a visit from an interviewer that would administer the questionnaire and collect a saliva sample. The individuals were informed that they were free to decline participation, and that they could find out more about the study with a telephone call or email; the contact information was provided in the letter. Participants that could not be located after several attempts (at different times of the day and different days of the week) and those that declined to participate in the study were replaced at random. The replacements were chosen from eligible individuals of the same sex, within a 5-year age group, and within the same district of residence. Substitutions accounted for 50.7% and 54.6% of the pre- and post-legislation surveys, respectively. Individuals that agreed to participate were interviewed at home by trained interviewers. Participants were asked to sign an informed consent form before proceeding with the face-to-face interview. In case of subjects aged 16 an 17, parental written consent was obtained. The same questionnaire was used in both surveys (on traditional paper in the pre-legislation survey and in computer-assisted form in the post-legislation survey). Additional questions were included in the second survey regarding the smoke-free legislation. The questionnaire included information on socio-demographics, tobacco consumption, self-assessed exposure to SHS in different settings (at home, work/educational venues, during leisure time, and in public and private transportation vehicles), and attitudes toward smoking restrictions. After completing the questionnaire, respondents were asked to provide a sample of saliva for the cotinine analysis, and weight and height were measured. The Research and Ethics Committee of Bellvitge University Hospital approved the study protocols and the informed consent forms, including parental written consent.

### Self-reported SHS exposure of non-smokers

Non-smokers were defined as individuals that, at the time of the interview, reported that they did not smoke, and they had a salivary cotinine concentration ≤10 ng/mL [17]. This group included individuals that had never smoked and ex-smokers.

*Exposure to SHS at home* was determined with two questions: “Currently, how many individuals per day usually smoke inside your home?” and “During the past week, how many cigarettes (per day) have been smoked in your presence inside your home?” Answers were gathered for typical working and non-working days. Based on these two questions, we derived a dichotomous variable of exposure to SHS at home: (1) non-exposed individuals, which included those with no exposure according to answers to both questions, and (2) exposed individuals, which included all others. *Exposure to SHS at work or an education venue* was determined with two questions: “Does anybody smoke in close proximity to you at work?” and “How many hours per day do you think you are exposed to tobacco smoke at your education venue?” We also derived a dichotomous variable of exposure to SHS at the workplace and/or education venue: (1) non-exposed individuals, which included those with no exposure according to answers to both questions, and (2) exposed individuals, which included all others. *Exposure to SHS at leisure time* was determined with the question “How much time have you spent in any place with tobacco smoke that was not home or work?” The answers were gathered for typical working and non-working days. For analysis, we derived a dichotomous variable of exposure to SHS during leisure time: (1) non-exposed individuals, which included those with no exposure according to the answer to the question, and (2) exposed individuals, which included all others. *Exposure to SHS at public and private transportation* was determined with two questions: “During the last week, were you in a public transportation vehicle while someone was smoking?” and “During the last week, were you in a private transportation vehicle while someone was smoking?” Based on these two questions, we derived a dichotomous variable of exposure to SHS in public and private transportation vehicles: (1) non-exposed individuals, which included those with no exposure according to answers to both questions, and (2) exposed individuals, which included all others. *Exposure to SHS in any setting* was defined as exposure in at least one of the above mentioned settings.

### Salivary cotinine

We asked the participants to provide a saliva sample to determine the cotinine levels. Cotinine is the main metabolite of nicotine; it is a stable, specific, sensitive biomarker of tobacco smoke in biological fluids, with a half-life of 15–17 h, and it reflects SHS exposure in the last 5–7 days[18]. We followed the same protocol in both surveys for collecting the saliva sample[6], [16]. Briefly, participants were asked to rinse their mouths and then suck on a lemon candy (Smint^R^) to stimulate saliva production. They were asked to provide about 9 mL of saliva by spitting into a funnel placed in a test tube. The sample was separated into 3 mL aliquots and frozen at −80°C for storage. The frozen samples were sent to the Bioanalysis Research Group of IMIM (Hospital del Mar Medical Research Institute) in Barcelona. Salivary samples from the pre-legislation survey were analyzed in 2007 with gas chromatography followed by mass spectrometry detection (GC/MS). The limit of quantification was 1 ng/mL and the limit of detection was 0.3 ng/mL. Salivary samples from the post-legislation survey were analyzed in 2012 with liquid chromatography coupled with tandem mass spectrometry (LC/MS/MS) with multiple reaction monitoring. The limit of quantification was 0.1 ng/mL and the limit of detection was 0.03 ng/mL; the quantification error was <15%. Because the latter method was more sensitive and had a lower limit of quantification than the former method, all available saliva samples from the pre-legislation survey with cotinine concentrations below 1 ng/mL (n = 245) were reanalyzed in 2012 with the LC-MS/MS method. The values from the second analysis were used in the statistical analysis. To determine the reliability of cotinine values from the pre-legislation survey, 41 saliva samples with previous values between 1 and 10 ng/mL were chosen at random, and cotinine was assessed with the LC/MS/MS. This analysis showed very low variation (less than +/− 1 ng/mL) in the concentration values obtained with both methods of analysis.

### Statistical analysis

We calculated prevalence rates (%) and 95% confidence intervals (CI) for exposure to SHS among non-smokers in the different settings. Results were stratified by sex, age (16–44, 45–64, and ≥65 years), and educational level (less than primary and primary school, secondary school, and university). The data were fitted with multivariate log-binomial models to assess the prevalence ratios (PR) and 95% CI of exposure to SHS among non-smokers before and after the implementation of the legislation. The models were adjusted for sex, age, and educational level. Geometric means (GM) and geometric standard deviations (GSD) were computed to describe the cotinine concentrations among non-smokers, due to its skewed distribution[17], [19]. The data were fitted with generalized linear regression models of the log-transformed salivary cotinine concentration, adjusted for potential confounders. We also estimated the percentage changes in salivary cotinine concentration by comparing the geometric mean of the concentrations before and after the legislation. Samples with values below the limit of detection were assigned a value of 0.05 ng/mL (half the limit of detection value). Statistical analyses were performed with SPSS v17.0 and Stata 10.

## Results

### Sample

**Figure 1:**
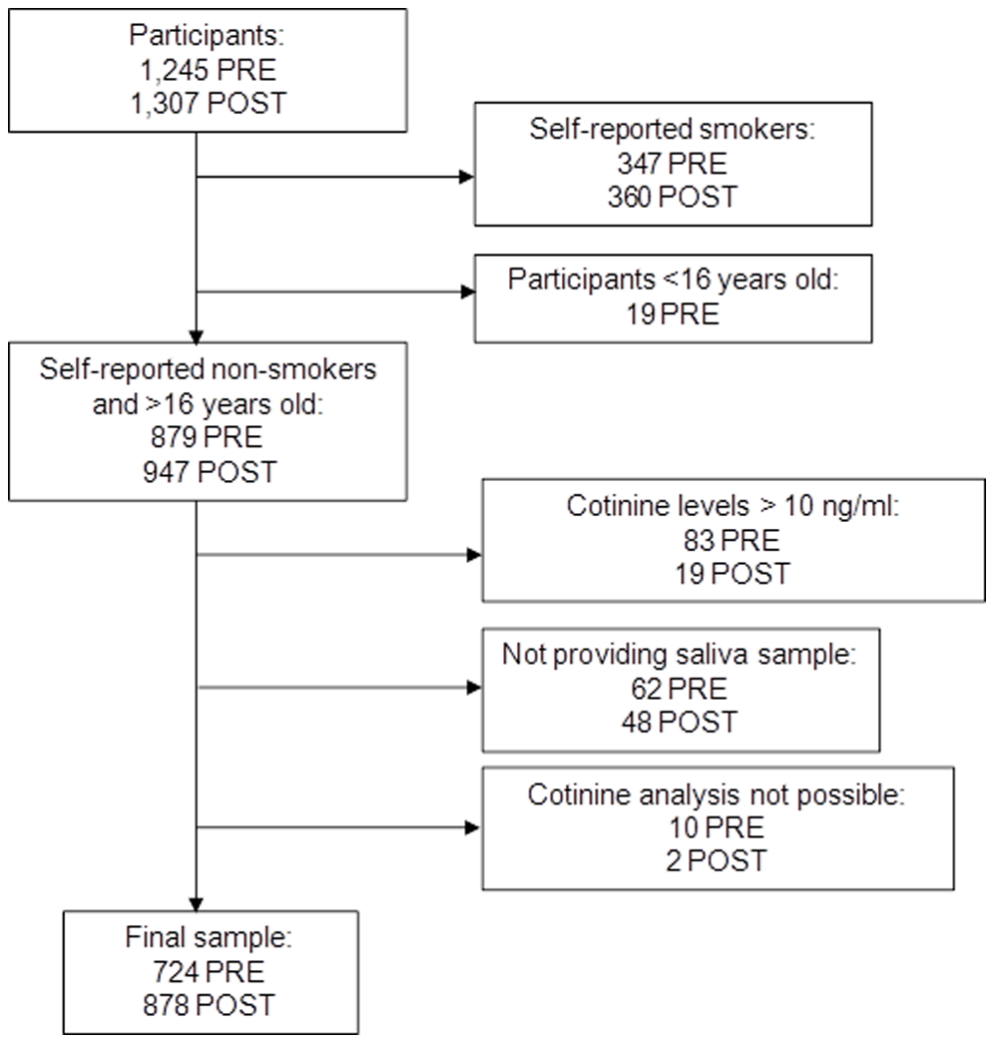
Flow chart with the sample selection in both surveys (PRE: 2005–06 and POST: 2011–12) and exclusions from the initial sample. Footnote to Figure 1. From the initial sample in each survey, we excluded people who declared to be smokers and people <16 years old. Among people who declared to be non-smokers, we excluded those with unreliable cotinine levels for non-smokers (this is, they had smoked at the time of the interview). We also excluded people who did not provide the saliva sample or in which the cotinine analysis was not possible because of insufficient sample or technical error.

A total of 2,552 participants were interviewed; 1,245 subjects were in the pre-legislation survey and 1,307 were in the post-legislation survey. The samples were similar in the proportions of men and women, but we found significant differences in age and educational level. 879 (70.6%) participants in the pre-legislation survey and 947 (72.5%) participants in the post-legislation survey were self-reported non-smokers. Of the non-smokers, 110 (62 in the pre-legislation and 48 in the post-legislation surveys) were not included in the analysis, because they did not provide a saliva sample; in addition, 12 (10 in the pre-legislation and 2 in the post-legislation survey) were excluded, because cotinine analysis was not possible (i.e., insufficient sample). 83 non-smokers from the pre-legislation survey and 19 from the post-legislation survey were excluded, because they had cotinine concentrations consistent with active smoking (>10 ng/mL). Therefore, the final sample for analysis included a total of 1602 non-smokers; 724 (58.2% of those interviewed) before the legislation and 878 (67.2% of those interviewed) after the legislation (Figure 1).

### Changes in self-reported exposure to SHS

The prevalence of self-reported exposure to SHS in any setting fell from 75.7% in 2004–05 to 56.7% in 2011–12 (relative reduction −25.1, p<0.001) (Table 1); this included reduced exposures in the home, from 32.5% to 27.6% (−15.1%, p<0.05); at work/education venue, from 42.9 to 37.5 (−12.6%, p = 0.11); during leisure time, from 61.3% to 38.9% (−36.5%, p<0.001); and in public transportation vehicles, from 12.3% to 3.7% (−69.9%, p<0.001). Overall, the prevalence of SHS exposure declined more sharply among women than among men (29.2% vs. 19.4%, p<0.001). Non-smoking adults between 45 and 64 years old showed the greatest reduction in the prevalence of SHS exposure (−34.3%, p<0.001); the prevalence in adults aged 65 years or older was reduced by 25.6% (p<0.001), and the prevalence in adults between 16 and 44 was reduced by 24.6% (p<0.001) (Appendix S1). The prevalence of exposure to SHS was reduced to a similar extent for individuals with different educational levels (Appendix S1). After controlling for sex, age, and educational level, self-reported exposure to SHS in any setting after the legislation was significantly reduced (PR: 0.46; 95%CI: 0.40 to 0.54), including at home, at work/educational venues, during leisure time, and in public transport vehicles (Table 1).

**Table 1:** Self-reported exposure to secondhand smoke in non-smokers before (2004–05) and after (2011–12) the smoke-free legislation, Barcelona, Spain; results are stratified by setting.

Self-reported exposure to secondhand smoke	n	% of non-smokers exposed (95% CI)	Prevalence ratio* (95% CI)
**Any setting** **			
Before the legislation	720	75.7 (72.6–78.8)	1
After the legislation	871	56.7 (53.4–60.0)	0.46 (0.40 to 0.54)
**Home** **			
Before the legislation	721	32.5 (29.1–35.9)	1
After the legislation	878	27.6 (24.6–30.6)	0.78 (0.65 to 0.94)
**Work/education venues** **			
Before the legislation	364	42.9 (37.8–48.0)	1
After the legislation	507	37.5 (33.3–41.7)	0.79 (0.63 to 0.98)
**Leisure time** **			
Before the legislation	723	61.3 (57.7–64.9)	1
After the legislation	872	38.9 (35.7–42.1)	0.38 (0.32 to 0.44)
**Public transportation** **			
Before the legislation	626	12.3 (9.7–14.9)	1
After the legislation	669	3.7 (2.3–5.1)	0.26 (0.16 to 0.41)
**Private transportation** **			
Before the legislation	585	9.4 (7.0–11.8)	1
After the legislation	616	10.7 (8.3–13.1)	0.97 (0.67 to 1.41)

*Based on multivariate log-binomial models, adjusted for sex, age, and educational level.

**The figures do not sum the total because of missing values.

### Changes in salivary cotinine levels

**Figure 2:**
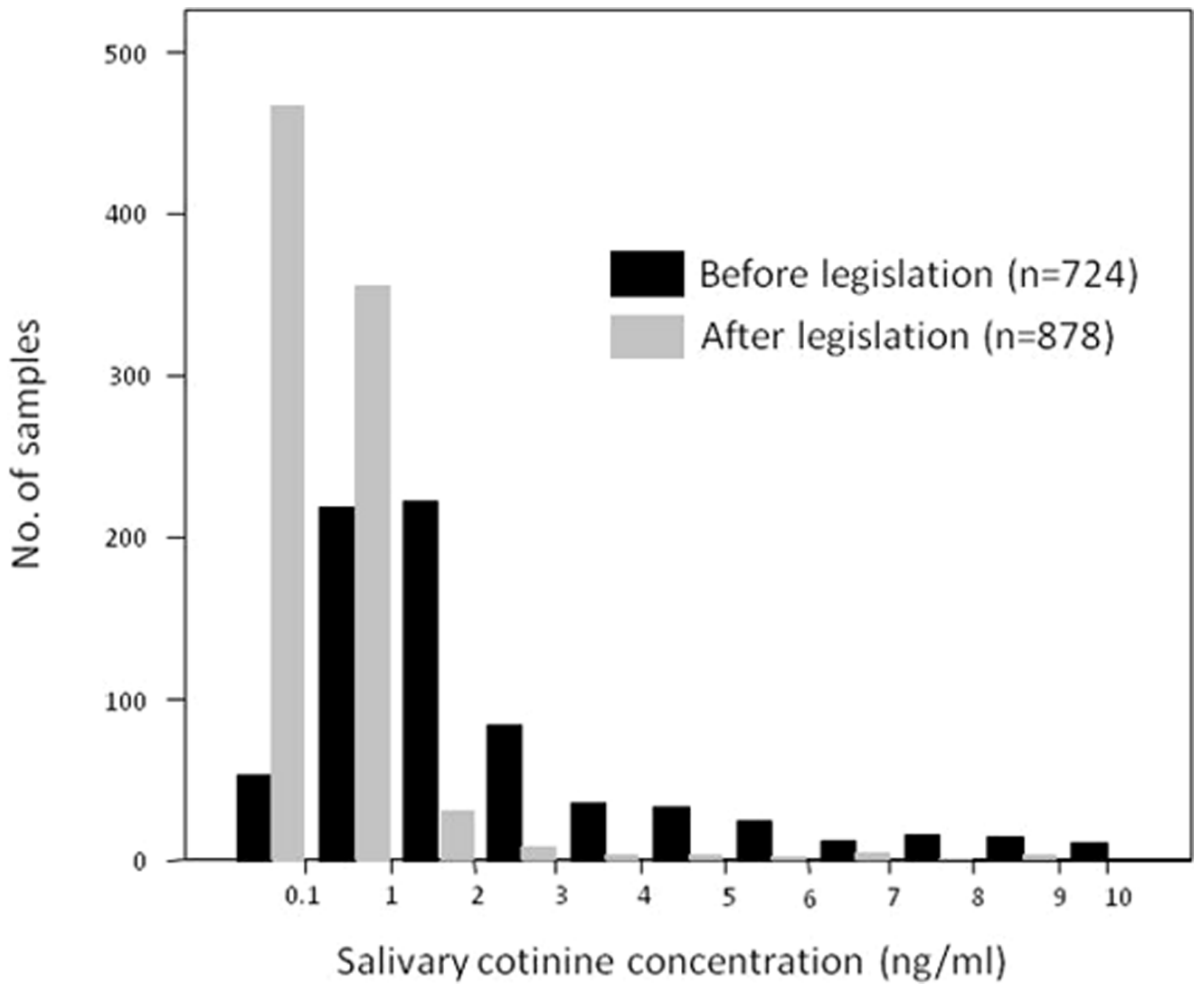
Distribution of salivary cotinine concentrations (ng/mL) among the non-smoker adult population, before (2004–05) and after (2011–12) the smoke-free legislation, in Barcelona, Spain.

Figure 2 shows the distribution of cotinine values among the non-smokers before and after legislation. The proportion of non-smokers with cotinine concentrations below the quantification limit (0.1 ng/mL) increased from 7.3% (53 samples) before the legislation to 53.2% (467 samples) after the legislation.

Table 2 compares the geometric mean values of salivary cotinine concentrations before and after the legislation among non-smokers. The results are stratified according to socio-demographic variables. The geometric mean of the cotinine concentrations among all adult non-smokers fell from 0.93 ng/mL before the legislation to 0.12 ng/mL (p<0.001) after the legislation. After adjusting for sex, age, and educational level, the reduction in cotinine concentration was 87.6% (p<0.001). The adjusted reduction in cotinine concentration after the implementation of the law was similar for participants of all ages. However, adult non-smokers with a university education showed the greatest adjusted reduction in cotinine concentration (Table 2).

**Table 2:** Change in the geometric means of salivary cotinine concentrations (ng/mL) before (2004–05) and after (2011–12) the smoke-free legislation, Barcelona, Spain; results are stratified according to socio-demographic variables.

	Before legislation		After legislation		Percentage of change* (95% CI)
	N	GM (GSD) (ng/mL)	N	GM (GSD) (ng/mL)	
**All subjects**	724	0.93 (4.01)	878	0.12 (3.12)	87.6 (76.7–102.0)
**Sex**					
Men	296	1.11 (3.65)	380	0.12 (2.91)	89.4 (80.6–102.1)
Women	428	0.82 (4.22)	498	0.12 (3.28)	86.1 (74.4–102.7)
**Age (years)** **					
16–44	236	1.00 (3.66)	361	0.12 (3.09)	88.0 (78.1–102.7)
45–64	234	0.82 (4.17)	254	0.13 (3.18)	85.4 (73.9–104.1)
≥65	251	0.98 (4.19)	263	0.11 (3.10)	89.2 (80.6–102.9)
**Educational level** **					
Less than primary and primary	342	0.87 (4.16)	236	0.12 (3.27)	86.1 (79.4–103.5)
Secondary	132	0.97 (3.95)	341	0.14 (3.28)	85.2 (73.7–104.3)
University	249	0.98 (3.83)	300	0.10 (2.75)	90.2 (82.2–102.1)

GM: Geometric mean.

GSD: Geometric standard deviation.

*Based on the adjusted geometric mean derived from a generalized linear model that included all the variables in the table.

**The figures do not sum the total because of missing values.

## Discussion

This was the first study to evaluate using both self-reports and a personal biomarker of exposure to SHS the impact of the stepped Spanish smoke-free legislation (laws 28/2005 and 42/2010) on SHS exposure in different settings among adult non-smokers from the general population. We found that self-reported exposure to SHS and salivary cotinine levels significantly decreased after the implementation of the legislation. This reduction was observed at workplaces, during leisure time, and even in settings not regulated by the law, like in the home and public transportation.

### Self-reported second-hand smoke exposure

The reduction in SHS exposure between 2004–05 and 2011–12 was greater for women than men and for individuals aged 45 to 64 compared with other age groups. Haw and Gruer[20] also evaluated changes in self-reported exposure to SHS among adult non-smokers after the implementation of smoke-free legislation in Scotland. They found that, after legislation, self-reported SHS exposure fell for all the settings assessed. Similarly, we observed a 25.1% reduction in SHS exposure among participants exposed in any setting. However, we are not able to distinguish the effects of the first (28/2015) and second (42/2010) bans on the reductions observed. Previous evaluations of the 28/2005 law showed important reductions in the exposure to SHS at the workplace[8], but that law did not affect the exposure to SHS at home or during leisure time[9], [11] nor in bars or restaurants[8], [10]. In the present study, the highest reductions in self-reported SHS exposure were observed in public transportation vehicles and during leisure time.

Data from another study in Spain showed that both airborne nicotine and PM2.5 decreased by more than 90% in bars and restaurants after the implementation of law 42/2010[14]. At the population level, a reduction in the self-reported exposure to SHS during leisure time after 2010 has been also oberved in Galicia[11]. Those results and the results obtained in the present study demonstrated the importance of the new legislation (Law 42/2010), which extended the prohibition of smoking to all hospitality venues without exception. These venues were places where young, adult non-smokers were mostly exposed during their leisure time. We also observed a significant relative reduction (15.1%) in the home, which confirmed no displacement of smoking to this setting but an unexpected positive side-effect of the smoke-free legislation. This finding agreed with other previous studies performed at the individual level[20]–[24] and at the ecological level[25]. We found a 12.6% reduction in self-reported exposure to SHS at work and educational venues. Previous studies in Spain[9], [11] showed greater reductions in self-reported exposure at work between 2005 and 2006. However, our results were consistent with another study,[5] which showed that 39.8% of non-smokers were exposed to SHS at work and educational venues after the implementation of Law 28/2005 (which prohibited smoking in the workplace, but not hospitality venues).

### Cotinine concentrations

The proportion of non-smokers that had undetectable cotinine concentrations increased from 7.3% before the 28/2005 law to 53.2% after the implementation of the 42/2010 law. Our results confirmed the positive impact of smoke-free laws on SHS exposure at the population level. For example, after legislation, in New York, Bauer et al.[26] found an increase in the proportion of respondents with cotinine concentrations below the detection limit (from 32.5% to 52.4%); in Scotland, Haw and Gruer[20] also observed an increase in individuals with undetectable cotinine (from 11.3% to 27.6%); and, in England, Sims et al.[27] found that the odds of having undetectable cotinine were 1.5 times higher than before the legislation.

In addition to this shift in the distribution of the non-smoking population towards lower levels of cotinine, the mean concentration declined from 0.93 ng/mL to 0.12 ng/mL (adjusted reduction of 87.6%). This reduction in cotinine concentration was greater than those obtained after the implementation of smoke-free legislation in New York[26], Scotland[20], and England[27] (reductions of 47%, 39%, and 27%, respectively). The larger decrease in Spain might be explained by the fact that the salivary cotinine concentrations among non-smokers in our study (0.93 ng/mL) before the 28/2005 legislation was 2 to 9 times higher than salivary cotinine concentrations obtained in New York[26], England[27], and Scotland[20] before the smoke-free bans (0.078 ng/mL, 0.14 ng/mL, and 0.43 ng/mL, respectively); the post-legislation concentrations were similar in the four different populations. In the absence of smoke-free legislation, the higher salivary cotinine levels in Spain among non-smokers (higher SHS exposure) could be explained by the higher prevalence of smoking in the population. After the implementation of smoke-free legislation, SHS exposure would decrease, regardless of the prevalence of smoking.

### Strengths and limitation of the study

One potential limitation of the study was an information bias derived from the use of a questionnaire. Self-reported, adult non-smokers represented 70.6% of the participants interviewed in the pre-legislation survey and 72.5% in the post-legislation survey. These prevalences were consistent with data from the 2006 and 2011 Spanish National Health Interview Surveys (Ministerio de Sanidad y Consumo: Encuesta Nacional de Salud 2006, 2013). This limitation was reduced by using an objective, specific biomarker of SHS exposure, and by asking the participants about their exposure in both private and public places, including the home, work/educational venues, leisure venues, and transportation vehicles. Thus, we covered the primary settings where SHS exposure can occur.

Another limitation is that we did not have data after the first law and previous to the second law, thus preventing us to elucidate the separate effects of both laws, as would have been of great interest given the stepped nature of the Spanish smoke-free legislation. However, the interpretation of our results together with the previous studies focused on the first law allows to globally evaluating the effects of the Spanish smoke-free laws.

This was a repeated cross-sectional study, which was potentially more likely to be biased than a longitudinal study. However, longitudinal studies can be subject to some bias, due to the loss of participants in the follow-up, which reduces its advantages. Nevertheless, repeated cross-sectional surveys that include a biological marker have been shown to be a valid method for evaluating smoke-free legislation[18], [28], [29].

This study included representative, random samples of the population of Barcelona (Spain) and it evaluated the impact of smoke free legislation on exposure to SHS with a combination of self-reported exposure and cotinine as an objective biomarker of SHS exposure. To minimize differences between the two collection periods, we used the same strategy in collecting the pre and post legislation data. Additionally, the fieldwork was performed during different days of the week, including weekends, and in different months to avoid systematic biases due to potential seasonal and timing aspects of data collection. The method for analyzing cotinine in the post legislation survey was more sensitive and had a lower limit of quantification than that used in the pre legislation survey. However, we reanalyzed the samples in the pre-legislation survey with the new method, and found satisfactory agreement in the results. Individuals that declined to participate were replaced at random with individuals with the same characteristics to prevent problems with sample size and selection biases. Although we had a high percentage of substitutions in both surveys, we obtained a high percentage of non-smokers that provided saliva samples in the pre- and post- legislation surveys (92.9% and 94.9%, respectively); this proportion was higher than those observed in similar assessments in Scotland (64.8% and 63.1%, respectively) [20] and in New York (33%, overall)[26].

## Conclusions

This study showed that the implementation of a stepped smoke-free legislation (laws 28/2005 and 42/2010) was accompanied by a large reduction in SHS, both self-reported and assessed by means of salivary cotinine levels, in the adult non-smoking population in Barcelona, Spain. The strategy of strengthening Law 28/2005 to hospitality venues without exceptions was clearly effective. We observed a high reduction in SHS exposure during leisure time, and a reduction in SHS exposure at home contrary to the speculative tobacco industry hypothesis of displacement of smoking from public to private places. Based on the results of this study, comprehensive tobacco control policies were effective in reducing SHS exposure. Thus, over time, the law will result in a reduction in morbidity and mortality among nonsmoking adults.

## Supporting Information

Appendix S1 **Prevalence of self-reported exposure to secondhand smoke in non-smokers measured before (2004-05) and after (2011–12) the smoke-free legislation, Barcelona, Spain; results are stratified by sex, age, educational level, and settings.**
(DOCX)

Checklist S1 **STROBE 2007 (v4) Statement—Checklist of items that should be included in reports of cross-sectional studies.**
(DOCX)
