# Supplementary material for: Impact of the Spanish Smoke-Free Legislation on Adult, Non-Smoker Exposure to Secondhand Smoke: Cross-Sectional Surveys before (2004) and after (2012) Legislation
Source: PLoS One. 2014 Feb 27;9(2):e89430. doi: 10.1371/journal.pone.0089430 (PMC3937341; doi:10.1371/journal.pone.0089430)
Supplement: Appendix S1 — Prevalence of self-reported exposure to secondhand smoke in non-smokers measured before (2004-05) and after (2011–12) the smoke-free legislation, Barcelona, Spain; results are stratified by sex, age, educational level, and settings. (DOCX) [file pone.0089430.s001.docx]

**Appendix S1. Prevalence of self-reported exposure to secondhand smoke in non-smokers measured before (2004-05) and after (2011-12) the smoke-free legislation, Barcelona, Spain; results are stratified by sex, age, educational level, and settings**

|  | **Exposed in any setting** | | | **Exposed at home** | | | **Exposed at work/education venues** | | | **Exposed during leisure time** | | | **Exposed in public transport vehicles** | | | **Exposed in private transport vehicles** | | |
| --- | --- | --- | --- | --- | --- | --- | --- | --- | --- | --- | --- | --- | --- | --- | --- | --- | --- | --- |
|  | % before | % after | PR | % before | % after | PR | % before | % after | PR | % before | % after | PR | % before | % after | PR | % before | % after | PR |
|  |  |  |  |  |  |  |  |  |  |  |  |  |  |  |  |  |  |  |
| **All** | 75.7 | 56.7 | 0.59 | 32.5 | 27.6 | 0.82 | 42.9 | 37.5 | 0.84 | 61.3 | 38.9 | 0.52 | 12.3 | 3.7 | 0.29 | 9.4 | 10.7 | 1.15 |
|  |  |  |  |  |  |  |  |  |  |  |  |  |  |  |  |  |  |  |
| **Sex** |  |  |  |  |  |  |  |  |  |  |  |  |  |  |  |  |  |  |
| Men | 74.2 | 59.8 | 0.67 | 26.9 | 22.1 | 0.80 | 42.7 | 41.3 | 0.96 | 63.5 | 42.7 | 0.55 | 13.3 | 3.7 | 0.27 | 7.3 | 8.3 | 1.15 |
| Women | 76.7 | 54.3 | 0.54 | 36.3 | 31.7 | 0.85 | 43.0 | 33.8 | 0.74 | 59.7 | 36.0 | 0.49 | 11.7 | 3.8 | 0.31 | 11.0 | 12.7 | 1.17 |
|  |  |  |  |  |  |  |  |  |  |  |  |  |  |  |  |  |  |  |
| **Age (years)** |  |  |  |  |  |  |  |  |  |  |  |  |  |  |  |  |  |  |
| 16-44 | 96.6 | 72.8 | 0.39 | 35.3 | 25.8 | 0.68 | 47.1 | 44.2 | 0.92 | 90.3 | 57.8 | 0.37 | 19.9 | 5.7 | 0.26 | 15.9 | 12.5 | 0.77 |
| 45-65 | 81.6 | 53.6 | 0.45 | 38.0 | 29.5 | 0.73 | 37.3 | 26.9 | 0.67 | 63.2 | 31.0 | 0.37 | 10.1 | 1.5 | 0.15 | 7.8 | 10.4 | 1.36 |
| ≥65 | 50.8 | 37.8 | 0.67 | 24.9 | 28.1 | 1.15 | - | - | - | 32.4 | 20.6 | 0.59 | 7.0 | 3.1 | 0.43 | 4.0 | 7.9 | 2.04 |
|  |  |  |  |  |  |  |  |  |  |  |  |  |  |  |  |  |  |  |
| **Educational level** |  |  |  |  |  |  |  |  |  |  |  |  |  |  |  |  |  |  |
| Less than primary and primary | 64.2 | 44.7 | 0.58 | 33.6 | 28.4 | 0.81 | 42.1 | 33.3 | 0.74 | 44.3 | 25.5 | 0.50 | 9.8 | 3.0 | 0.30 | 6.9 | 7.1 | 1.03 |
| Secondary | 87.1 | 62.2 | 0.47 | 42.4 | 34.6 | 0.77 | 43.8 | 44.3 | 1.02 | 78.0 | 45.0 | 0.39 | 14.2 | 5.1 | 0.34 | 14.6 | 13.9 | 0.95 |
| University | 85.1 | 60.2 | 0.48 | 25.3 | 19.0 | 0.72 | 42.8 | 32.5 | 0.70 | 75.9 | 42.6 | 0.39 | 14.5 | 2.6 | 0.17 | 9.9 | 9.2 | 0.93 |

% prevalence

PR: prevalence ratio
